# Particular evolution of newborns exposed to HIV/AIDS in the Romanian cohort

**DOI:** 10.1186/1471-2334-13-S1-P3

**Published:** 2013-12-16

**Authors:** Mariana Mărdărescu, Adrian Streinu-Cercel, Cristina Petre, Ruxandra Neagu-Drăghicenoiu, Rodica Ungurianu, Sorin Petrea, Ana Maria Tudor, Alina Cibea, Delia Vlad, Carina Matei, Marieta Iancu, Alexandra Mărdărescu, Ioana Anca, Cosmina Cristea, Mihai Mitran

**Affiliations:** 1 National Institute for Infectious Diseases “Prof. Dr. Matei Bals”, Bucharest, Romania; 2 Carol Davila University of Medicine and Pharmacy, Bucharest, Romania; 3 Romanian HIV/AIDS Centre, National Institute for Infectious Diseases “Prof. Dr. Matei Balş”, Bucharest, Romania; 4 Institute for Mother and Child Protection “Alfred Rusescu”, Bucharest, Romania; 5 Clinical Hospital for Obstetrics and Gynecology “Prof. Dr. Panait Sârbu”, Bucharest, Romania

## Background

HIV infection in Romania has always stood for a particular event compared to Western and Central Europe epidemics. The specificity of the Romanian epidemic lays in a high rate of incidence in children, registered at the end of 1980s and beginning of 1990s, otherwise known as the “epidemiological accident”. Over 70% of people living with HIV/AIDS (PLWHA) in Romania are long term survivors. The main objectives of our prospective study were to assess the incidence of congenital malformations in children born to HIV positive mothers from the Romanian cohort of long-term survivors; to determine whether an increase in this incidence is correlated with: HIV infection per se, the length of HIV infection, antiretroviral therapy (ART), use of “so called” legal drugs (ethnobotanics), HIV+ART, HIV+ART+ethnobotanics.

## Methods

In two different groups of patients we assessed the following parameters: gestational age, weight at birth, Apgar score, clinical development, ultrasound exams at birth and at 18 months (Aloka Pro Sound SSD-550 SV).

## Results

The first study group comprised 150 children to 485 HIV positive mothers (35% of all children evaluated in the time span: 2000-2011); the control group was made up of 75 children to non-HIV mothers (4% of all children admitted at the Institute for Mother and Child Protection “Alfred Rusescu”, Bucharest).

In the study group, 64 children (42.66% of the exposed children) were diagnosed with different malformations. The most prevalent was atrial septal defect but there were several complex cardiac malformations. Out of the 75 children in the control group, 14 malformations (19.73%) and 11 cardiac malformations (15%) were detected. A first statistical evaluation revealed that the incidence of cardiac malformations in the exposed children group is not statistically different to the control group (p=0.11), the complexity grade being higher. A statistically significant difference is observable for the other types of malformations (p<0.01).

## Conclusion

The impact of antiretroviral treatment and HIV infection on the fetus and newborn represents a continuously debated topic throughout the world. However, one cannot exclude the duration of the mother’s disease, especially for Romania’s epidemic particularities, that are to be further studied. Within this context, in order to better comprehend the evolution of mother to child transmission, a program has recently been launched, namely The National Registry for the surveillance of HIV pregnant women and perinatally exposed newborns.

